# Correction: Trends, and patterns, of premarital sexual intercourse and its associated factors among never-married young women aged 15–24 in Sierra Leone

**DOI:** 10.1371/journal.pone.0327679

**Published:** 2025-07-02

**Authors:** Augustus Osborne, Castro Ayebeng, Peter Bai James, Camilla Bangura, Richard Gyan Aboagye, Bright Opoku Ahinkorah

The comma after trends and patterns should be omitted in the article title. The correct title is: Trends and patterns of premarital sexual intercourse and its associated factors among never-married young women aged 15–24 in Sierra Leone. The correct citation is: Osborne A, Ayebeng C, James PB, Bangura C, Aboagye RG, Ahinkorah BO (2024) Trends and patterns of premarital sexual intercourse and its associated factors among never-married young women aged 15–24 in Sierra Leone. PLoS ONE 19(8): e0309200. https://doi.org/10.1371/journal.pone.0309200
